# Ferroptosis in neurodegenerative diseases: mechanisms and therapeutic potential of stem cell derivatives

**DOI:** 10.3389/fcell.2025.1577382

**Published:** 2025-03-21

**Authors:** Ting Zhang, Yusu Zhang, Jinpeng Xie, Dandan Lu, Lihong Wang, Shuaifei Zhao, Jing Zhou, Yang Cheng, Ting Kou, Jue Wang, Ying Chen, Lei Xu, Xiangyu Hu, Yuxiu Ying, Jun Wang, Xiaoshuang Xin, Xu Xu, Siyun Lei, Chenyu Qiu, Jinhua Wu, Qiqi Lyu, Tong Cao

**Affiliations:** ^1^ Oujiang Laboratory (Zhejiang Lab for Regenerative Medicine, Vision and Brain Health), School of Pharmaceutical Science, Wenzhou Medical University, Wenzhou, Zhejiang, China; ^2^ School and hospital of Stomatology, Wenzhou Medical University, Wenzhou, Zhejiang, China; ^3^ Department of Stomatology the Second Affiliated Hospital of Xinjiang Medical University, Urumqi, China; ^4^ Oujiang Laboratory (Zhejiang Lab for Regenerative Medicine, Vision and Brain Health), Wenzhou, Zhejiang, China; ^5^ Department of Neuroradiology, Singapore General Hospital, Singapore, Singapore

**Keywords:** ferroptosis, neurodegenerative diseases, aging, stem cell derivatives, neuroprotection

## Abstract

Ferroptosis, a non-apoptotic, iron-dependent form of regulated cell death, is closely related to the pathogenesis of neurodegenerative diseases. Stem cells and their derivatives exhibit remarkable potential in modulating ferroptosis, offering promising therapeutic intervention for neurodegenerative diseases. In this review, we systematically explore neurological aging and its association with cognitive impairment and neurodegenerative diseases, with focus on the molecular mechanisms of ferroptosis in neurodegenerative diseases and the potential therapeutic strategies of stem cell derivatives for neurological diseases.

## 1 Introduction

Aging is a biological process marked by a gradual decline in physiological function, impacting almost every organ system. It serves as a primary risk factor for numerous age-related diseases (Alrouji et al., 2024; Campisi et al., 2019). Epidemiological data indicate that 11% of the global population is currently aged 60 or above, with this figure projected to increase to 22% by 2050 (Kanasi et al., 2000). Neurodegenrative diseases, whose prevalence rises with aging, pose a significant challenge to public health. For example, approximately 1 in 10 adults age 65 or above are affected by Alzheimer disease (AD) (Miller and O'Callaghan, 2003), which is irreversible and currently incurable (Hou et al., 2019; Heemels, 2016).

Aging is influenced by a variety of external and internal factors (López-Otín et al., 2023). Mitochondrial dysfunction and the disruption of proteostasis have been implicated in the development of chronic inflammation, alongside the accumulation of misfolded proteins, oxidative damage, glycation, and ubiquitination, collectively leading to neuronal injury and cell death (López-Otín et al., 2023). Age-associated dysfunctions in the central nervous system are closely associated with the initiation and progression of neurodegenerative disorders, such as AD, Parkinson’s disease (PD), and amyotrophic lateral sclerosis (ALS) (Park et al., 2020). Ongoing research into the mechanisms of brain aging is crucial for developing strategies to slow disease progression, ultimately enhancing brain health and improving the quality of life for the aging population.

Iron plays a crucial role in maintaining normal physiological functions and is especially vital for neural development and neuroprotection during infancy and childhood (Zeidan et al., 2021; Georgieff, 2017). Studies have shown that iron deficiency in brain tissue before the age of three can result in long-term neural dysfunction (Zeidan et al., 2021; Georgieff, 2017). Iron, the most prevalent transition metal in the brain, plays a crucial role in sustaining the brain’s elevated metabolic demands, supporting myelination, and facilitating the synthesis of neurotransmitters like dopamine and norepinephrine (Dev and Babitt, 2017). Nevertheless, iron also has the potential to catalyze the generation of reactive oxygen species (ROS), thereby exacerbating intracellular oxidative stress and damaging cellular macromolecules (Killilea et al., 2003). Extensive research has demonstrated that excessive iron deposition in brain tissue can negatively impact brain function, particularly in older adults (Dev and Babitt, 2017). While the most common clinical symptom of low iron levels in adults is anemia, elevated iron concentrations can be highly toxic to cells (Dev and Babitt, 2017). Iron overload in the brain induces oxidative damage to astrocytes and neuronal toxicity, thereby promoting neurodegenerative diseases (Dev and Babitt, 2017). Such pathological iron deposition may stem from dysregulated intracellular iron homeostasis or age-related physiological alterations (Killilea et al., 2003; Masaldan et al., 2018). For example, impaired ferritin phagocytosis in senescent cells may alter cellular iron acquisition and storage (Killilea et al., 2003; Masaldan et al., 2018). Iron accumulation in aging tissues has been well-documented, contributing to increased oxidative stress and cellular dysfunction, which further promotes the accumulation of senescent cells (Killilea et al., 2003; Masaldan et al., 2018). Therefore, a close interrelationship may exist between the loss of iron homeostasis and cellular senescence (Killilea et al., 2003; Masaldan et al., 2018). Iron overload enhances lipid peroxidation and ROS production in mitochondria, potentially triggering ferroptosis (Chen et al., 2022).

Ferroptosis is a non-apoptotic, iron-dependent form of regulated cell death (RCD), marked by excessive lipid peroxidation, disruptions in cellular metabolism and redox balance (Jiang et al., 2021). Modulation of ferroptosis to alleviate the progression of various organ injuries and degenerative diseases has become a key area of research. Stem cells, recognized for their capacity for self-renewal and multipotent differentiation, have emerged as a promising therapeutic strategy in recent years. The use of stem cells and their derivatives in treating degenerative diseases and facilitating tissue repair holds significant potential. Currently, there is growing interest in investigating the regulation of ferroptosis by stem cell derivatives. The subsequent sections will offer an in-depth analysis of the relationship between neurodegenerative diseases and ferroptosis, alongside the role of stem cell-derived products in modulating ferroptosis.

## 2 Aging of nervous system

Neurological aging refers to the gradual decline and alterations in nervous system function that occur with advancing age. As the aging process progresses, the metabolic functions of neurons deteriorate, potentially leading to reduced efficiency in neural transmission. In certain brain regions, such as the hippocampus, neuronal loss may also occur. Additionally, changes in neurotransmitter levels can affect cognitive function. The study of cognitive aging has a long history, with foundational work dating back to the 1960s. For instance, Welford and Birren’s seminal article, “Behavior, Aging, and the Nervous System” (Welford and Birren, 1965), laid the groundwork for cognitive aging theory. Over the past 5 decades, research has increasingly focused on cognitive neuroscience, particularly the age-related changes in brain structure and function. A growing body of evidence now underscores the close relationship between cognitive function and aging (Anderson and Craik, 2017). Although the connection between aging and cognitive decline remains a topic of debate, some studies suggest that physiological aging may not necessarily lead to cognitive impairment. For example, the ‘Scaffolding Theory of Aging and Cognition’ (STAC), proposed in 2009, integrates extensive behavioral research and neuroimaging data, highlighting the brain’s ability to maintain functionality through self-reorganization and repair. This plasticity allows the brain to adapt to aging (Park and Reuter-Lorenz, 2009). However, despite this adaptive capacity, brain function still declines with aging, and there are significant individual differences in the brain’s ability to reorganize functionally. Aging is a complex process shaped by both genetic and environmental factors. Epigenetic changes play a pivotal role in the aging process (Traxler et al., 2023). These changes encompass a range of mechanisms, including DNA methylation, histone modification, and non-coding RNA regulation, all of which modulate gene expression to influence cellular functions and behaviors (Traxler et al., 2023). Environmental factors, such as diet, lifestyle, and exposure to harmful substances, can significantly impact epigenetic marks. For instance, unhealthy dietary habits and a sedentary lifestyle may result in alterations in DNA methylation patterns, which in turn can affect gene expression and cellular function (Park and Reuter-Lorenz, 2009; Traxler et al., 2023). When adverse factors disrupt the brain’s homeostatic repair mechanisms, physiological aging may progress to pathological aging, ultimately leading to neuronal loss and neurodegeneration (Park and Reuter-Lorenz, 2009; Traxler et al., 2023). In fact, while many forms of aging are adaptive in early life, the ongoing transition of cells to a senescent state is linked to tissue dysfunction and age-related diseases (Traxler et al., 2023).

### 2.1 Neurodegenerative diseases

Neurodegenerative diseases refer to a category of disorders marked by the gradual degeneration or loss of neuronal function. These conditions typically affect motor skills, cognitive abilities, and the capacity to perform everyday activities. Notable examples of such conditions include AD, PD, ALS, Huntington’s disease (HD), and multiple sclerosis (MS). Pathological aging, which involves the gradual loss of cellular homeostasis and a decline in the capacity for adaptive responses, is strongly associated with cognitive impairment and the onset of neurodegenerative diseases. In recent years, there has been an increasing urgency to investigate the mechanisms driving the onset and progression of brain diseases, with the aim of extending healthy lifespan and addressing age-related neurodegenerative disorders. It is well established that aging of the nervous system is closely linked to cellular senescence, which may serve as a key pathophysiological factor in neurodegenerative diseases. For instance, McNamara et al. observed that the depletion of microglia exacerbates myelin pathology, suggesting that the integrity of myelin becomes increasingly reliant on healthy microglia as aging progresses (McNamara et al., 2023). This finding indicates that microglial dysfunction may lead to myelin damage, a process closely tied to aging and neurodegenerative disease development. As a result, microglia are considered a promising therapeutic target for conditions involving myelin dysfunction, such as aging and neurodegenerative diseases (McNamara et al., 2023). Beyond microglia, other brain cells—such as neurons, astrocytes, and oligodendrocytes—are also critical to cognitive function and are significantly impacted by aging, cellular senescence, and the progression of neurodegenerative diseases. The decline in the function and health of these cells contributes to the gradual deterioration of cognitive abilities. Understanding the roles of these cells in both aging and disease, and exploring the underlying mechanisms, is crucial for developing effective preventive or therapeutic strategies.

### 2.2 Ferroptosis in the nervous system

Iron is abundantly present in the brain, and due to the protective nature of the blood-brain barrier, iron homeostasis within the brain is largely independent of that in other tissues. The uptake and transport of iron in the brain are regulated through interactions between endothelial cells and perivascular astrocytes. Transferrin-bound iron (Tf-Fe (3+)) binds to transferrin receptor 1 (TfR1) on the luminal surface of endothelial cells, forming the Tf-Fe (3+)-TfR1 complex. This complex is subsequently internalized via endocytosis. Within the acidic environment of the endosome, iron is released from transferrin, and Fe (3+) is reduced to Fe (2+) by the action of Steap3 (six-transmembrane epithelial antigen 3) on the endosomal membrane. Fe (2+) is then transported into the cytoplasm through the divalent metal transporter 1 (DMT1) (Yoshida, 2012). Once in the cytoplasm, iron can be utilized for the biosynthesis of iron-sulfur clusters (Fe-S) and heme in mitochondria, stored in ferritin, or enter the labile iron pool (LIP) for immediate cellular use. The transport of ferritin in the extracellular matrix also involves several mechanisms. For example, ferritin can be oxidized from Fe (2+) to Fe (3+) by GPI-anchored ceruloplasmin located on the end feet of astrocytes. The resulting Fe (3+) binds to transferrin in the brain interstitial fluid, and the Tf-Fe (3+) complex can then be absorbed by neurons (Yoshida, 2012). Dysregulation of iron metabolism and abnormal iron accumulation are linked to a variety of hereditary, and non-hereditary neurological diseases. As a redox-active metal, iron can generate harmful free radicals if its intracellular levels are not tightly regulated. Age-related changes in cellular and molecular processes governing iron metabolism can lead to iron homeostasis disorders and iron deposition, which in turn may trigger ferroptosis. Due to the extensive membrane phospholipid surface area of neurons, these cells are particularly vulnerable to lipid peroxidation during oxidative stress, making them highly susceptible to ferroptosis. Under conditions of iron and lipid metabolism imbalance, the accumulation of iron and subsequent lipid peroxidation can lead to neuronal dysfunction and cell death (David et al., 2022; Jacquemyn et al., 2024).

## 3 Ferroptosis-related mechanisms in nervous system aging

**FIGURE 1:**
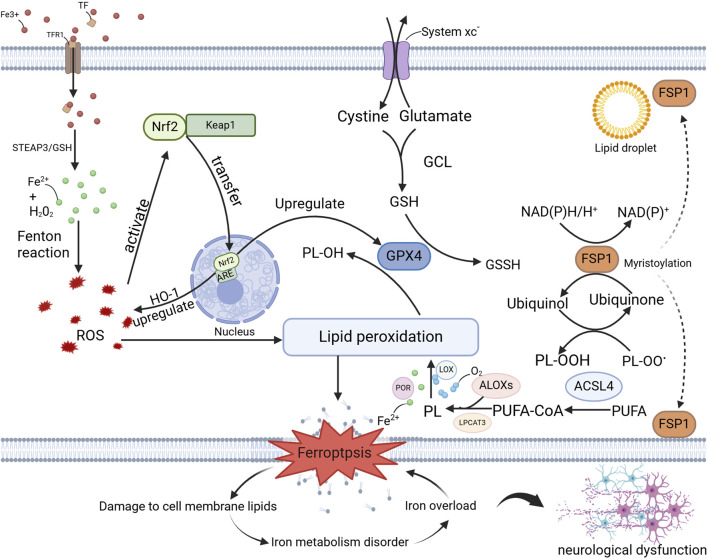
Mechanisms of Ferroptosis in the Nervous System. This illustration delineates the molecular mechanisms of ferroptosis in neurodegenerative diseases, encompassing iron ion metabolism, oxidative stress, lipid peroxidation, ferroptosis itself, and its impact on cellular membrane lipids, iron homeostasis, and neurological function. The key molecules and pathways involved—namely, the GPX4 pathway, the FSP1 pathway, and the Nrf2 pathway—provide crucial insights into the role of ferroptosis in neurodegenerative diseases and offer potential targets for the development of novel therapeutic strategies, as detailed in the preceding text. Abbreviations: TFR1, Transferrin Receptor 1; TF, Transferrin; STEAP3, Six-transmembrane epithelial antigen of the prostate 3; ROS, eactive Oxygen Species; PL, Phospholipid; PL-OH, Phospholipid Hydroxide; PL-OOH, Phospholipid Hydroperoxide; OH-1, Heme Oxygenase-1; GCL, Glutamate-Cysteine Ligase; GSH, Glutathione; GSSH, Glutathione Persulfide; LOX, Lipoxygenase; POR, Phospholipid Oxidation Reductase; LPCAT3, Lysophosphatidylcholine Acyltransferase 3; PUFA-CoA, Polyunsaturated Fatty Acid-Coenzyme A; PUFA, Polyunsaturated fatty acid.

The concept of programmed cell death dates back to the early 1960s, describing a regulated form of cell death that occurs in the absence of any exogenous interference, maintaining cellular homeostasis (Hirschhorn and Stockwell, 2019). This genetically regulated biological process is crucial for maintaining organismal homeostasis, and its disruption can lead to the onset of various pathological conditions (Hirschhorn and Stockwell, 2019). While the phenomenon of iron-dependent cell death has been recognized in numerous studies, it was not until 2012 that this process was formally named ferroptosis (Hirschhorn and Stockwell, 2019). Unlike other forms of programmed cell death, ferroptosis is a pathological mode of cell death defined by the excessive accumulation of iron-dependent lipid ROS. This form of cell death is linked to the dysfunction of multiple organs. In the nervous system, the unique sensitivity of neurons to ferroptosis makes it particularly relevant to neurodegenerative diseases (Jiang et al., 2021; Jacquemyn et al., 2024). Exploring the mechanisms of ferroptosis in the context of aging in the nervous system provides important insights that may guide the development of targeted therapies for neurodegenerative diseases. Figure 1 summarizes relevant mechanisms of ferroptosis in the nervous system (Jiang et al., 2021).

### 3.1 Iron-dependent lipid peroxidation

Ferroptosis is marked by excessive accumulation of iron-dependent lipid peroxides, especially the peroxidation of polyunsaturated fatty acids (PUFAs). The concept of ferroptosis was first introduced by Dixon, J. S., and Lemberg, M. K. in 2012 (Dixon et al., 2012). Their studies, using fluorescent probes such as H2DCFDA and C11-BODIPY, revealed that treatment of HT-1080 cells with erastin (a known ferroptosis inducer) led to the accumulation of cytoplasmic and lipid ROS within 2 h, ultimately resulting in cell death after 6 h. This observation highlighted a close relationship between cell death and lipid peroxidation (Dixon et al., 2012).

From a mechanistic standpoint, several lipid-related regulatory factors have been identified, including glutathione peroxidase 4 (GPX4), prostaglandin-endoperoxide synthase 2 (PTGS2), ferritin heavy chain 1 (FTH1), solute carrier family seven member 11 (SLC7A11), carnitine palmitoyltransferase 1 A (CPT1A), and lipoxygenase ALOX5 (Chen et al., 2024; Ma et al., 2024; Li K. et al., 2023). These factors play essential roles in lipid oxidation processes. Modulation of these key regulatory factors or the use of antioxidants to suppress lipid peroxidation within cells can effectively inhibit ferroptosis (Chen et al., 2024; Ma et al., 2024; Li K. et al., 2023).

Iron plays a pivotal role in the generation of hydroperoxides from membrane phospholipids (PLs) and can also produce electrophilic products by cleaving the weak O-O bonds of these hydroperoxides (Dixon et al., 2012; Sun et al., 2021). These products are considered the key mediators of ferroptosis. Moreover, the introduction of exogenous iron can amplify cell death, highlighting the pivotal role of iron in the generation of ROS (Dixon et al., 2012; Sun et al., 2021). This process can be attenuated by the use of iron chelators, such as deferoxamine (DFO), which regulate intracellular iron levels and reduce ROS accumulation, thereby mitigating ferroptosis and subsequent cell death (Dixon et al., 2012; Sun et al., 2021).

### 3.2 Oxidative stress and antioxidant system imbalance

Oxidative stress and disruptions in the antioxidant system are key factors in the initiation of ferroptosis, especially in the context of neurodegenerative diseases, metabolic disorders, and other pathological conditions. For instance, several well-known ferroptosis inducers, such as erastin and RSL3, function as inhibitors of the antioxidant system. Although ferroptosis represents a pathological form of cell death, it is a regulated process that can be modulated by various signaling pathways. In the following sections, we will explore several key signaling pathways that regulate ferroptosis by influencing the antioxidant system.

#### 3.2.1 GPX4 pathway

Glutathione (GSH) and GPX4 play crucial roles in cellular biology and the antioxidant defense system. GPX4, a key enzyme in this system, exerts a potent inhibitory effect on ferroptosis by facilitating the clearance of ROS (Shen et al., 2023; Wei et al., 2023). Depletion of GSH is closely associated with reduced GPX4 levels, resulting in an imbalance in the antioxidant system and the accumulation of toxic phospholipid hydroperoxides. In the presence of iron ions, this process promotes the onset of ferroptosis (Shen et al., 2023; Wei et al., 2023). Thus, GSH serves as an important countermeasure against lipid peroxidation. As a central regulator of ferroptosis, GPX4 has garnered significant attention in medical research, particularly in the context of neurodegenerative diseases, where it holds considerable therapeutic potential. Upregulation of GPX4 expression can inhibit ferroptosis, providing protection to neurons from its damaging effects. For instance, Thonningianin A, a novel ferroptosis inhibitor, activates the AMPK/Nrf2 pathway to enhance Nrf2 nuclear translocation and GPX4 expression (Yong et al., 2024). This mechanism effectively suppresses ferroptosis and demonstrates notable neuroprotective effects in both cellular models and *Caenorhabditis elegans* models of AD (Yong et al., 2024). Furthermore, adeno-associated virus (AAV)-mediated overexpression of GPX4 has been shown to significantly alleviate cognitive dysfunction, particularly in spatial learning and memory, induced by traumatic brain injury (TBI), offering promising new therapeutic avenues for TBI treatment (Fang et al., 2023).

Furthermore, ferroptosis represents a “double-edged sword,” with significant potential in cancer therapy (Xie et al., 2024). Inhibition of GPX4 has been shown to induce cancer cell death. Kruppel-like factor 11 (KLF11) is a member of the Kruppel-like zinc finger transcription factor family, which is involved in a variety of physiological and pathological processes, including cell proliferation, differentiation, and apoptosis (Zhao et al., 2023). Studies have utilized Chromatin Immunoprecipitation Sequencing (ChIP-Seq) and RNA Sequencing (RNA-Seq) to identify GPX4 as a downstream target of KLF11 (35). KLF11 binds to the promoter region of GPX4 and inhibits its transcription, thereby reducing GPX4 expression (Zhao et al., 2023). By modulating the GPX4-GSH axis, KLF11 promotes ferroptosis in lung adenocarcinoma cells, thereby inhibiting cell proliferation and enhancing chemosensitivity (Zhao et al., 2023). These findings suggest that KLF11 may serve as a potential therapeutic target to enhance the efficacy of chemotherapy through the regulation of ferroptosis. Future research could further explore the role of KLF11 in other types of cancer and its potential therapeutic applications, thereby fully realizing its potential in the field of antitumor treatment.

#### 3.2.2 FSP1 pathway

The FSP1-CoQ10 pathway was first identified in a 2019 study by K. Bersuker et al., who observed substantial variability in the sensitivity of various cancer cell lines to GPX4 inhibitors (Bersuker et al., 2019). This led to the hypothesis that additional factors might regulate resistance to ferroptosis. Using CRISPR/Cas9-based synthetic lethal screening, they identified ferroptosis suppressor protein 1 (FSP1, formerly known as AIFM2) as a potent factor promoting ferroptosis resistance. Notably, FSP1 is a key component of a parallel ferroptosis suppression pathway that operates independently of GPX4. FSP1 utilizes NADH as a coenzyme to reduce coenzyme Q (CoQ) to its reduced form. This reduced form of CoQ functions as a lipophilic radical-trapping antioxidant (RTA), inhibiting the propagation of lipid peroxidation. Furthermore, inhibition of COQ2, the enzyme responsible for CoQ synthesis, or genetic knockout of COQ2, significantly increases cellular sensitivity to ferroptosis inducers. FSP1 can also suppress ferroptosis by modulating CoQ levels, providing an independent mechanism of antioxidant protection when GPX4 activity is compromised. These findings establish FSP1 as a critical factor in maintaining cellular redox balance, underscoring its potential applications in cancer therapy and the treatment of oxidative stress-related diseases (Bersuker et al., 2019).

#### 3.2.3 ACSL4 and ALOXs pathways

Acyl-CoA synthetase long-chain family member 4 (ACSL4) and lipoxygenases (ALOXs) are two enzymes implicated in lipid metabolism, both acting as key promoters of ferroptosis. ACSL4 catalyzes the esterification of long-chain PUFAs, thereby increasing the availability of oxidizable substrates, while ALOXs convert these substrates into lipid hydroperoxides. The combined actions of these enzymes significantly elevate intracellular levels of lipid hydroperoxides, creating the necessary conditions for ferroptosis. ACSL4 facilitates the generation of lipid hydroperoxides through the esterification of PUFAs, which are critical drivers of ferroptosis. Upregulation of ACSL4 expression promotes the accumulation of lipid hydroperoxides, thereby triggering ferroptosis (Ma et al., 2024; Huang et al., 2024; Cui et al., 2021a). Inhibition of ACSL4 activity can effectively suppress ferroptosis. The expression and activity of ACSL4 are regulated by multiple signaling pathways, including p53 and Nrf2. For example, p53 promotes ferroptosis by suppressing SLC7A11 expression while simultaneously upregulating ACSL4, thereby enhancing lipid peroxidation (Song et al., 2023). Qian Huang et al. identified AS-252424 (AS), a potent ferroptosis inhibitor, through a kinase inhibitor library screen (Huang et al., 2024). AS directly targets and binds to the glutamine 464 (Q464) site of ACSL4, inhibiting its enzymatic activity and subsequently reducing lipid peroxidation and ferroptosis (Huang et al., 2024). In microglia, ACSL4 promotes inflammatory responses, and its high expression is also associated with increased sensitivity of tumor cells to ferroptosis. Thus, ACSL4 inhibitors hold potential therapeutic value in treating neurodegenerative diseases and cancer (Ma et al., 2024; Zhou et al., 2023).

In Huntington’s disease models, ALOX5 has been identified as a key factor in HTT-Q94-induced, ACSL4-independent ferroptosis, emphasizing its independent role in ferroptosis, particularly in neurodegenerative diseases (Song et al., 2023). ALOXs interact closely with GPX4 and SLC7A11; upregulation of ALOXs diminishes the protective effects of GPX4 and impairs SLC7A11 function, thereby promoting ferroptosis (Li K. et al., 2023; Song et al., 2023). Claulansine protects dopaminergic neurons from ferroptosis by inhibiting the nuclear translocation of ALOX5 and reducing the production of toxic lipids, such as 5-HETE. Moreover, ALOX5 is a critical factor in Huntington’s disease, and its inhibition has been shown to significantly improve behavioral deficits and reduce neuronal damage. These findings elucidate the molecular mechanisms underlying ferroptosis regulation, offering a foundation for novel therapeutic strategies in neurodegenerative diseases (Li K. et al., 2023; Song et al., 2023).

#### 3.2.4 Nrf2 pathway

Nrf2 (nuclear factor erythroid 2-related factor 2) is a transcription factor that regulates the expression of a wide array of antioxidant and cytoprotective genes. The Nrf2 signaling pathway plays a critical role in modulating cellular antioxidant responses and maintaining redox homeostasis, thereby influencing the regulation of ferroptosis. The mechanism through which Nrf2 regulates ferroptosis likely involves interactions with multiple signaling pathways. In neurons, Nrf2 primarily protects cells from ferroptosis-induced damage by activating antioxidant pathways, such as GPX4 (Li J. et al., 2023; Yang et al., 2023). For instance, vitamin D mitigates ferroptosis-induced damage to hippocampal neurons and improves cognitive function in aging mice. This protective effect is mediated by the activation of the Nrf2/HO-1 signaling pathway through the vitamin D receptor (VDR), thereby inhibiting ferroptosis (Li J. et al., 2023). Similarly, salidroside (SAL), a natural compound with neuroprotective and immunomodulatory properties, has been shown to activate the Nrf2/GPX4 axis, suppress neuronal ferroptosis, and reduce CD8^+^ T cell infiltration, leading to improved cognitive function in SAMP8 mice (Yang et al., 2023). These studies highlight potential therapeutic targets for ferroptosis-related diseases. Future research should focus on further elucidating the specific mechanisms by which the Nrf2 signaling pathway operates in various neurodegenerative diseases and evaluating the safety and efficacy of pharmacological interventions aimed at modulating this pathway. Additionally, long-term activation of Nrf2 and its therapeutic implications should be thoroughly investigated to optimize treatment outcomes.

## 4 Ferroptosis and neurodegenerative diseases

In recent years, the role of ferroptosis in neurodegenerative diseases has become more clearly defined, particularly in conditions such as AD, PD, and HD. This section provides a summary and analysis of the current understanding of the relationship between ferroptosis and neurodegenerative diseases.

### 4.1 Alzheimer’s disease

The pathogenesis of AD is multifactorial, involving a complex interplay of various pathophysiological mechanisms. The traditional amyloid cascade hypothesis posits that the abnormal accumulation of amyloid-beta (Aβ) peptides is the primary initiating event in AD, leading to subsequent tau protein hyperphosphorylation, neuronal apoptosis, and progressive cognitive decline (Scheltens et al., 2021). However, as research into the pathophysiology of AD has progressed, this hypothesis has been significantly refined and expanded. Recent studies have shown that a preclinical cellular phase, lasting several years, precedes the onset of clinical symptoms in AD (Scheltens et al., 2021). This phase is characterized by early alterations in neurons, microglia, and astrocytes, which occur before Aβ deposition and interact with Aβ accumulation to collectively drive disease progression (Scheltens et al., 2021). This process is closely linked to disruptions in iron homeostasis. As previously discussed, elevated levels of labile iron promote the generation of lipid peroxides, which are highly cytotoxic and further exacerbate neuronal dysfunction (Dev and Babitt, 2017).

Ferroptosis, a regulated form of cell death, plays a significant role in the initiation, progression, and outcomes of AD. A deeper exploration of the molecular mechanisms underlying ferroptosis will enhance the theoretical framework of AD pathogenesis and offer novel insights for early diagnosis and intervention. In both AD mouse models and AD patients, the expression of the iron export protein ferroportin 1 (Fpn) is downregulated, leading to ferroptosis and memory deficits (Bao et al., 2021). Ferroptosis inhibitors have been shown to reduce amyloid-beta (Aβ) aggregation-induced neuronal death and memory impairment, while restoring Fpn expression ameliorates ferroptosis and improves cognitive function in AD mice (Bao et al., 2021). Moreover, studies investigating ferroptosis-related mechanisms in AD have identified several compounds with potential therapeutic applications, including vitamin D, salidroside, artemisinin, and RSL3 (Li J. et al., 2023; Yang et al., 2023; Deng PX. et al., 2024; Cui et al., 2021b). As previously mentioned, vitamin D suppresses ferroptosis through the VDR/Nrf2/HO-1 signaling pathway, highlighting its potential as a protective agent in age-related neurodegenerative diseases (Li J. et al., 2023). Vitamin D supplementation may thus serve as a simple and safe preventive measure to enhance cognitive function and improve the quality of life in elderly populations (Li J. et al., 2023). Salidroside (SAL), a natural compound derived from “Rhodiola”species, has been shown to modulate the Nrf2/GPX4 axis and iron transport, reduce Aβ and iron deposition, and alleviate neuronal damage and cognitive deficits (Yang et al., 2023). These neuroprotective effects offer promising therapeutic strategies for neurodegenerative diseases, particularly AD (Yang et al., 2023). Artemisinin, an antimalarial drug with anti-inflammatory and antioxidant properties, has recently been found to inhibit neuronal ferroptosis by targeting KEAP1 to activate the Nrf2-SLC7A11-GPX4 pathway, thereby improving cognitive dysfunction in AD mouse models (Deng PX. et al., 2024). This discovery provides new mechanistic insights into the potential of artemisinin as an AD therapeutic. RSL3, a multifunctional small-molecule compound traditionally known as a potent ferroptosis inducer, has shown unexpected dual roles. Recent studies demonstrate that RSL3 not only induces ferroptosis but also exhibits anti-inflammatory effects by upregulating Nrf2 protein expression, suppressing LPS-induced inflammatory responses, and enhancing ferroptosis resistance in microglia and macrophages (Cui et al., 2021b). This dual functionality suggests that RSL3 may have therapeutic potential in neurodegenerative diseases like AD. Collectively, these studies offer a solid theoretical and experimental foundation for developing innovative therapeutic strategies targeting ferroptosis in AD.

### 4.2 Parkinson’s disease

PD is a progressive neurodegenerative disorder characterized by the abnormal aggregation and pathological propagation of α-synuclein, with underlying mechanisms involving systemic dysfunction in mitochondrial, lysosomal, and nuclear functions. Studies of both genetic and sporadic cases have highlighted the complexity and heterogeneity of the disease (Morris et al., 2024). While genetic predisposition, aging, environmental factors, and oxidative stress are implicated in PD pathogenesis, the precise pathophysiological mechanisms remain incompletely understood (Morris et al., 2024). This complexity presents significant challenges for the development of effective therapeutic strategies. Current clinical treatments for PD primarily include anticholinergic agents (e.g., trihexyphenidyl) and dopaminergic agents (e.g., levodopa) (Balestrino and Schapira, 2020). While these medications can alleviate motor symptoms to some extent, they do not halt disease progression or reduce the long-term risk of disability in patients (Balestrino and Schapira, 2020). Therefore, a more thorough investigation into the pathophysiological mechanisms of PD—particularly its intricate molecular and cellular regulatory networks—holds critical scientific and clinical value for the development of novel anti-PD therapeutics.

The correlation between iron and PD has long been established (Dexter et al., 1989). Daily exposure to elevated iron levels is recognized as a risk factor for the development of PD (Dexter et al., 1992). Research has demonstrated that ferroptosis, an iron-dependent mode of cell death, is a critical contributor to the degeneration of dopaminergic neurons in PD (Lin et al., 2022). Ferroptosis is marked by the buildup of lipid peroxides and excessive iron accumulation, both of which are closely linked to the death of dopaminergic neurons in the substantia nigra pars compacta (SNc) of individuals with PD (Lin et al., 2022). In a study by A. Anandhan et al., overexpression of α-synuclein (α-syn) was found to increase neuronal susceptibility to ferroptosis by suppressing NRF2 protein levels (Anandhan et al., 2022). The loss of NRF2 exacerbates ferroptosis markers in PD-related brain regions, leading to neuronal loss and behavioral deficits. This suggests that a vicious cycle between α-syn and NRF2 inhibition may be a key mechanism driving the initiation and progression of PD, offering a novel, currently underexplored target for preventing disease onset and progression (Anandhan et al., 2022). Collectively, these studies highlight the critical role of ferroptosis in PD pathogenesis. Inhibiting ferroptosis or restoring the function of related proteins may offer promising strategies for slowing or halting PD progression.

Numerous studies have explored ferroptosis-related mechanisms to develop preventive and therapeutic strategies for PD. For example, FTH1 has been shown to inhibit ferroptosis in 6-hydroxydopamine (6-OHDA)-induced PD models by modulating ferritinophagy, a process that regulates intracellular iron levels (Tian et al., 2020). NADPH oxidase 4 (NOX4) may promote neuronal ferroptosis and neuroinflammation, contributing to dopaminergic neuron degeneration. Inhibition of NOX4 was found to alleviate behavioral deficits in PD model animals, reduce dopaminergic neuron loss, and suppress lipid peroxidation, suggesting that NOX4 could serve as a potential therapeutic target for PD (Lin et al., 2025). Claulansine exerts neuroprotective effects by interacting with the Ser663 site (a PKCα phosphorylation site) of ALOX5, thereby preventing its nuclear translocation and inhibiting ferroptosis in dopaminergic neurons (Li K. et al., 2023). Additionally, several other investigational compounds, including quercetin, morroniside, rapamycin, and iron chelators such as alpha-lipoic acid (ALA), have demonstrated neuroprotective properties by mitigating ferroptosis (Lin et al., 2022; Li M. et al., 2023; Liu et al., 2023; Zheng et al., 2023). These findings establish a mechanistic foundation for designing innovative therapeutic interventions targeting ferroptosis. Targeting ferroptosis-related pathways or molecules may offer promising avenues for slowing or halting the progression of PD.

### 4.3 Huntington’s disease

HD is an autosomal dominant inherited neurodegenerative disorder characterized by a distinct phenotype that includes chorea, dystonia, incoordination, cognitive decline, and behavioral disturbances. Typically manifesting in midlife, the disease follows a progressive course, with symptoms worsening over time and ultimately leading to devastating consequences for affected individuals and their families. The pathogenic mutation underlying HD is an expanded CAG trinucleotide repeat within the gene encoding the huntingtin protein, resulting in an elongated polyglutamine tract at the protein’s N-terminus. This mutation confers a toxic gain-of-function property to the huntingtin protein, although the precise pathophysiological mechanisms remain incompletely understood (Manyam et al., 1978; Fragassi et al., 1992).

In HD, regional and laminar-specific neuronal loss in the neostriatum, neurodegeneration of selected thalamic nuclei, involvement of the cerebellar cortex and deep cerebellar nuclei, and pathology across various brainstem nuclei collectively contribute to the pathophysiological features that characterize the clinical phenotype of HD (Rüb et al., 2015). Oxidative stress and neuroinflammation are prominent features observed in the brains of HD patients. Iron accumulation exacerbates oxidative stress, thereby accelerating lipid peroxidation and mitochondrial dysfunction, which in turn, exacerbate neuroinflammatory processes. Ultra-high-field 7T MRI studies have identified correlations between cerebral iron levels and neuroinflammatory metabolites in HD patients, suggesting interconnected roles for oxidative stress, ferroptosis, and neuroinflammation in the disease pathogenesis (van de Zande et al., 2023). Although no disease-modifying therapies for HD are currently available, multiple promising therapeutic approaches are under development. Recent advances in therapeutic research include gene-silencing techniques, targeted small-molecule strategies, and efforts to identify key biomarkers for HD, which are critical for validating these emerging treatments (Fragassi et al., 1992).

Ferroptosis is closely linked to the pathogenesis of HD. In the striatum of HD patients, altered expression of ferroptosis-associated genes impacts neuronal survival. For example, laduviglusib has been shown to exert neuroprotective effects by targeting the ALOX5-mediated ferroptosis signaling pathway in microglia, enhancing mitochondrial function, and preventing neuronal loss (Liu et al., 2024). Furthermore, ALOX5-mediated ferroptosis plays a critical role in HD, as ALOX5 serves as a primary effector of mutant huntingtin (mHTT)-driven ferroptosis, presenting a novel therapeutic target (Zhou et al., 2023). Further investigation into the specific mechanisms of ferroptosis in HD, along with the development of therapies targeting this process, holds significant potential for improving clinical outcomes. Understanding the role of ferroptosis in HD not only deepens our insights into the disease’s complex pathophysiology but may also provide pivotal clues for novel therapeutic strategies. Future studies will continue to explore the regulatory mechanisms of ferroptosis-related pathways in HD and how this knowledge can be harnessed to design more effective treatments, ultimately yielding substantial benefits for patients.

### 4.4 Amyotrophic lateral sclerosis

ALS is a debilitating and ultimately fatal neuromuscular disease marked by the progressive degeneration of both upper and lower motor neurons, resulting in the loss of muscle function. The prevalence of ALS rises with age, with the highest incidence observed in individuals between 60 and 79 years of age (Feldman et al., 2022; Grad et al., 2017). The global standardized incidence rate is 1.68 per 100,000, though significant geographic and demographic variations exist (Feldman et al., 2022; Grad et al., 2017). Neuropathological hallmarks of ALS include motor neuron loss, axonal degeneration, reactive gliosis, and other neurodegenerative features (Grad et al., 2017). Despite significant advances in research, the precise pathological mechanisms driving ALS are still not fully elucidated (Grad et al., 2017). Ferroptosis, a regulated form of cell death marked by lipid peroxidation, elevated iron levels, and GSH depletion, has been implicated in the pathogenesis of ALS (Wang D. et al., 2023). Recent studies have identified depletion of GPX4, a key inhibitor of ferroptosis and antioxidant enzyme, in *postmortem* spinal cords of both sporadic and familial ALS patients. This depletion is correlated with impaired Nrf2 signaling and dysregulation of glutathione synthesis and iron-binding proteins. Experimental studies have demonstrated that overexpressing human GPX4 in a novel line of BAC transgenic SOD1G93 A mice notably delayed the onset of the disease, enhanced motor function, and prolonged lifespan. These findings suggest that strategies aimed at mitigating ferroptosis, such as activating and upregulating the GPX4 pathway, may offer promising therapeutic avenues for ALS (Wang et al., 2022).

Furthermore, Gene Set Enrichment Analysis revealed significant enrichment of ferroptosis-related pathways in ALS transcriptomic data. SPY1, which is downregulated in ALS, exhibited a positive correlation with GCH1 expression (Wang D. et al., 2023). By modulating the GCH1/BH4 axis and TFR1 expression, SPY1 effectively suppressed ferroptosis, attenuating ALS onset and progression (Wang D. et al., 2023). These results highlight the pivotal role of ferroptosis in ALS and establish both a theoretical and experimental basis for the development of innovative therapeutic strategies. Currently, only two drugs—riluzole and edaravone—are approved in some countries to slow ALS progression (Feldman et al., 2022). Future studies should aim to better define the mechanisms of ferroptosis in ALS, thereby facilitating the development of more effective therapeutic approaches.

## 5 Regulatory role of stem cell derivatives in ferroptosis

Stem cells are fundamental biological entities present in most multicellular organisms, characterized by their capacity for self-renewal and differentiation into diverse cell types (Tian et al., 2023). These cells are essential for both embryonic development and the maintenance of tissue homeostasis in adults. Based on their differentiation potential, stem cells are classified into totipotent (e.g., zygotes), pluripotent (e.g., embryonic stem cells), and unipotent or multipotent (e.g., adult stem cells) categories (Tian et al., 2023). Stem cells are fundamental sources for cellular replenishment during both embryogenesis and adult tissue repair (Tian et al., 2023). Stem cells hold immense potential in medical applications, particularly in the fields of tissue repair and regenerative medicine. For example, stem cells or their derivatives can be used to regenerate tissues damaged by disease or injury, including bone regeneration following fractures, vision restoration in retinal disorders, spinal cord injury repair, and myocardial tissue regeneration after heart attacks (Jin, 2017; Lindvall and Kokaia, 2005). Of particular note is their promise in treating intractable neurological conditions, where transplantation of stem cell-derived products may replace cells lost due to disease or injury, offering the potential to restore brain function (Jin, 2017; Lindvall and Kokaia, 2005). This approach provides a theoretical foundation for transformative treatments of severe neurodegenerative diseases, such as Parkinson’s disease and stroke. In comparison to direct stem cell transplantation, therapies using stem cell derivatives offer distinct advantages in terms of safety, controllability, and applicability. Derivatives like exosomes or cytokines carry specific bioactive molecules that can precisely modulate tissue repair processes while minimizing off-target effects. These characteristics enhance the therapeutic specificity of these treatments, while also reducing the risks associated with uncontrolled cell proliferation or immune rejection.

### 5.1 Mechanisms of ferroptosis regulation by stem cell derivatives

Stem cell derivatives, particularly exosomes, have shown diverse mechanisms in regulating ferroptosis, including the delivery of miRNAs, modulation of key ferroptosis-related protein expressions, and the activation or inhibition of specific signaling pathways. These mechanisms are pivotal in both neurological and non-neurological diseases. For instance, exosomes derived from Bone Marrow Stem Cells (BMSCs) modified with miR-340–3p (MB-exos) downregulate the expression of METTL3, reduce N6-methyladenosine levels, and stabilize Heme Oxygenase 1 (HMOX1) mRNA. This action inhibits ferroptosis and promotes recovery in injured rat uteri (Xiao et al., 2024). This study not only offers a novel therapeutic strategy for endometrial injury but also underscores the potential of exosomes in intercellular communication and disease treatment. In a mouse model of liver fibrosis induced by C-C Motif Chemokine Ligand 4 (CCL4), exosomes derived from mesenchymal stem cells (MSCs) carrying miR-26a were shown to promote ferroptosis in hematopoietic stem cells (HSCs) and regulate SLC7A11 expression, thereby alleviating liver fibrosis (Cao et al., 2024). This research provides new insights into using exosomes as nanoscale drug carriers for treating liver fibrosis and offers experimental support for their future clinical applications.

Moreover, stem cell derivatives can regulate key ferroptosis-related proteins or signaling pathways, demonstrating significant potential in treating neurological diseases. For example, Yong Wang et al. identified CHAC1 as a critical gene promoting ferroptosis in ischemic stroke through analysis of the Gene Expression Omnibus (GEO) database (Wang Y. et al., 2023). MiR-760–3p targets CHAC1 and inhibits its expression. To further assess its impact on ferroptosis and neurofunction, researchers intranasally delivered miR-760-3p-enriched Adipose-Derived Stem Cell Exosomes (ADSC-Exo) to mouse brains. This approach significantly improved neurobehavioral function in ischemia/reperfusion (I/R) injury mice, reduced the expression of ferroptosis-related protein markers (e.g., ACSL4 and GPX4), and decreased lipid peroxidation products (e.g., Malondialdehyde) (Wang Y. et al., 2023).

Similarly, in a subsequent study, M2-type microglia were observed to transiently increase and then rapidly decrease during the acute phase of ischemic stroke, suggesting their high sensitivity to ferroptosis (Wang et al., 2024). Using ADSC-Exo as a carrier, researchers engineered exosomes targeting M2-type microglia (M2pep-ADSC-Exo) to explore their therapeutic potential in ischemic stroke (Wang et al., 2024). The findings indicated that ADSC-Exo could regulate the Atf3/Slc7a11 axis, reduce lipid peroxidation and iron accumulation, inhibit inflammatory microenvironments, and promote neuronal survival, significantly improving neurofunction in I/R-injured mice (Wang et al., 2024). These results demonstrate that exosomes possess low immunogenicity and good tissue penetration, making them ideal drug delivery systems. Additionally, ADSC-Exo, as a non-cellular therapeutic approach, avoids the risks of immune rejection and tumorigenesis associated with stem cell transplantation, offering a more convenient and feasible alternative for clinical applications. Furthermore, genetic engineering of exosomes can enhance their targeting ability and therapeutic efficacy, providing new strategies for clinical use (Wang Y. et al., 2023; Wang et al., 2024).

Currently, several similar studies are exploring the regulatory mechanisms and clinical application prospects (Table 1). Multiple studies have shown that stem cell derivatives, particularly exosomes, exhibit significant therapeutic effects in various neurological disease models, including intracerebral hemorrhage (ICH), ischemic brain injury (MCAO), and spinal cord injury (SCI). The diversity of the mechanisms involved offers a theoretical basis for developing personalized treatment strategies for different neurological diseases, providing new approaches for future therapies.

**TABLE 1:** Summary of application of stem cell derivatives for ferroptosis modulation.

Researcher	Stem cell derivative	Exosome Modification/engineering	Disease Models	Main Mechanism of Action	Therapeutic effect
Liu et al. (2022)	MSCs-Exo	NA	Delayed Neurocognitive Recovery (dNCR) model in aged mice	Activation of the SIRT1/Nrf2/HO-1 signaling pathway	Improving cognitive dysfunction in dNCR aged mice
Yang et al. (2024)	MSC-EVs	NA	ICH mouse model	miR-214-3p inhibits ferroptosis marker genes (e.g., GPX4 and ACSL4); activates Nrf2/HO-1 pathway	Improvement of cerebral edema and hemorrhagic focus resorption after cerebral hemorrhage
Shao et al. (2022)	MSCs-exo	Enrichment in MSCs-exo lncGm36569	Acute Spinal Cord Injury (ASCI) mouse model	lncGm36569 competitively adsorbs miR-5627-5p, thereby upregulating the expression of FSP1 through the miR-5627-5p/FSP1 axis	Increased Basso Mouse Scale (BMS) scores and improved neurological function in ASCI mice
Deng et al. (2024b)	MSCs-exo	MSCs-exo overexpression of SRC-3	MCAO mouse model	SRC-3-exo by decreasing lipid peroxidation products (e.g., MDA, LPO) and increasing antioxidant capacity (e.g., GSH/GSSG ratio, CAT activity)	Significantly reduced brain tissue water content and infarct volume
Shao et al. (2024)	Hypoxia preconditioning of exosomes of ADSCs origin	HExos enhanced by hypoxic preconditioning (2% O_2_) and circ-Wdfy3 overexpression	SCI mouse model	Circ-Wdfy3 adsorbs miR-423-3p and upregulates GPX4 via the miR-423-3p/GPX4 pathway	Significantly reduced Spinal Cord Injury induced apoptosis in spinal cord tissues
Fan et al. (2023)	BMSC-Exos	Enrichment of miR-367-3p in BMSC-Exos	Experimental autoimmune encephalomyelitis (EAE) mouse model	miR-367-3p/EZH2/SLC7A11/GPX4 Axis upregulation GPX4	Decreased clinical symptoms, inflammation, and demyelination in spinal cord of EAE mice

### 5.2 Impact of stem cell derivatives from different origins

Table 1 shows researches focusing on the use of MSC-derived exosomes to improve neurological diseases through the modulation of ferroptosis. Numerous studies have demonstrated that these exosomes can regulate ferroptosis through the delivery of specific miRNAs. For example, MSC-derived exosomes have been shown to modulate key signaling pathways associated with ferroptosis, such as the Nrf2/GPX4 and SIRT1/Nrf2/HO-1 pathways (Liu et al., 2022; Yang et al., 2024). These exosomes exhibit neuroprotective effects in various disease models, including ICH, ischemic brain injury, and SCI. They reduce cerebral edema, improve neurological function, and inhibit cell death associated with ferroptosis.

Although both BMSC-derived and ADSC-derived exosomes have shown potential in regulating ferroptosis, the number of studies investigating these derivatives is relatively limited (Shao et al., 2024; Fan et al., 2023). Further exploration of their therapeutic potential is needed in future research.

In addition to MSCs, neural stem cells, embryonic stem cells, and pluripotent stem cells offer significant therapeutic potential for neurodegenerative diseases (Sugaya and Vaidya, 2018; Bernal and Peterson, 2004; Izrael et al., 2025). However, there remains a substantial research gap regarding the impact of their derivatives on neurological function and the regulation of ferroptosis. Future studies should explore this area further to uncover the potential of these stem cell derivatives in modulating ferroptosis and providing neuroprotection. Such research could pave the way for developing more effective therapeutic strategies for treating neurological disorders.

## 6 Conclusions and future perspectives

Researches demonstrate that neurologic aging is accompanied by diminished neuronal metabolic function, reduced efficiency of neural transmission, and neuronal death, ultimately leading to cognitive decline. Ferroptosis, a form of iron-dependent, lipid peroxidation-driven pathological cell death, is closely associated with neurodegenerative diseases such as Alzheimer’s disease, Parkinson’s disease, Huntington’s disease, and amyotrophic lateral sclerosis. Its core mechanism involves the accumulation of lipid peroxides, with key regulatory factors including GPX4, FSP1, ACSL4, ALOXs, and Nrf2. Furthermore, stem cells and their derivatives (e.g., exosomes) exhibit remarkable potential in modulating ferroptosis and providing neuroprotection, achieved through the delivery of miRNAs, regulation of ferroptosis-associated protein expression, and modulation of signaling pathways. Ferroptosis is a crucial factor in the pathogenesis of neurodegenerative diseases, and a thorough investigation of its regulatory mechanisms provides a theoretical basis for the development of novel therapeutic strategies for these disorders. Current studies have identified that stem cell-derived products can regulate ferroptosis through multiple mechanisms, highlighting their substantial therapeutic potential for neurological diseases. Future studies should focus on clarifying the precise mechanisms of ferroptosis-related pathways in neurodegenerative diseases and evaluating the therapeutic potential of stem cell derivatives. Additionally, translating these insights into the design of targeted, effective therapeutic interventions to deliver substantial clinical benefits for patients remains a major challenge for the scientific community.
